# Key components influencing the sustainability of a multi-professional obstetric emergencies training programme in a middle-income setting: a qualitative study

**DOI:** 10.1186/s12913-021-06385-5

**Published:** 2021-04-26

**Authors:** Kiren Ghag, Rachna Bahl, Cathy Winter, Mary Lynch, Nayda Bautista, Rogelio Ilagan, Matthew Ellis, Isabel de Salis, Timothy J. Draycott

**Affiliations:** 1 Department of Women’s Health, The Chilterns, Southmead Hospital, Bristol, BS10 5NB UK; 2 University of Bristol, Bristol, UK; 3 St Michael’s Hospital, Bristol, UK; 4 Project HOPE, Cebu, Philippines

**Keywords:** Obstetric emergencies, multi-professional training, Sustainability, Middle-income setting, Implementation

## Abstract

**Background:**

Multi-professional obstetric emergencies training is one promising strategy to improve maternity care. Sustaining training programmes following successful implementation remains a challenge. Understanding, and incorporating, key components within the implementation process can embed interventions within healthcare systems, thereby enhancing sustainability. This study aimed to identify key components influencing sustainability of PRactical Obstetric Multi-Professional Training (PROMPT) in the Philippines, a middle-income setting.

**Methods:**

Three hospitals were purposively sampled to represent private, public and teaching hospital settings. Two focus groups, one comprising local trainers and one comprising training participants, were conducted in each hospital using a semi-structured topic guide. Focus groups were audio recorded. Data were analysed using thematic analysis. Three researchers independently coded transcripts to ensure interpretation consistency.

**Results:**

Three themes influencing sustainability were identified; *attributes of local champions*, *multi-level organisational involvement* and *addressing organisational challenges*.

**Conclusions:**

These themes, including potential barriers to sustainability, should be considered when designing and implementing training programmes in middle-income settings. When ‘scaling-up’, local clinicians should be actively involved in selecting influential implementation champions to identify challenges and strategies specific to their organisation. Network meetings could enable shared learning and sustain enthusiasm amongst local training teams. Policy makers should be engaged early, to support funding and align training with national priorities.

**Supplementary Information:**

The online version contains supplementary material available at 10.1186/s12913-021-06385-5.

## Background

Multi-professional obstetric emergencies training is one of the most promising strategies to improve global maternity care [1, 2]. Effective training has been associated with improved perinatal outcomes [3–5] as well as positive impacts upon organisational and human factors [6]. Improvement in knowledge and attitudes does not always translate into improved outcomes [7]. Sustainability of effective training programmes following successful implementation projects remains a challenge [8, 9]. Healthcare staff are often faced with competing initiatives and priorities, [10] and many initiatives are not sustained after the initial funding ends [11]. Understanding, and incorporating, key sustainability components into the implementation process could improve the implementation of local training programmes and embed them within healthcare systems, [12] reducing the burden of promising but short-lived interventions on limited resources and funding.

Successful implementation of obstetric emergencies training programmes in high, low and poor socioeconomic areas of middle income settings is well described [5, 13–20]. However, there are few data describing implementation in middle-income settings, where a significant proportion of the world’s births occur. Middle-income settings have specific challenges, as despite a modest level of resource and income they often experience persistently high mortality and morbidity; hence findings from high and low-income settings may not be directly generalisable to middle-income settings. Interventions should be adapted to individual contexts for successful implementation [21] and therefore a better understanding of middle-income settings is required.

PRactical Obstetric Multi-Professional Training (PROMPT) is a multi-professional obstetric emergencies training programme with robust evidence of effect [13–16]. Local PROMPT courses are one-day, in-house training courses for multi-professional maternity staff, comprising lectures and practical scenarios focusing on emergencies such as pre-eclampsia, haemorrhage and shoulder dystocia. However, there is minimal experience of implementing PROMPT in middle-income settings despite successful implementation of PROMPT in a variety of high and low-income contexts internationally. The Philippines PROMPT Project was developed to address the needs of maternity care in the Philippines, a middle-income setting. Women birth in a range of public and private healthcare settings, including home births with or without the presence of skilled birth attendants, community birthing centres, rural health units, first level referral hospitals and tertiary hospitals. Maternal and neonatal mortality rates have declined in the Philippines in recent years (currently 120:100,000 live births and 12.6:1000 live births respectively) [22, 23] but failed to meet the targets set by the Millennium Development Goals [24]. There is an ambition at both local and government levels to continue to improve perinatal outcomes and progress towards achieving the Sustainable Development Goals for perinatal health [25, 26].

The Philippines PROMPT Project was a feasibility study investigating implementation of local PROMPT courses in seven pilot tertiary hospitals. The selection criteria for these hospitals included: tertiary urban hospitals, varied birth rates and a mix of public and private hospitals. The PROMPT training package was adapted to local clinical practices and needs following reflexive feedback from multidisciplinary teams [27]. Local PROMPT training was successfully implemented between December 2015 and February 2017: 31 local PROMPT courses delivered training to 816 multi-professional staff, 87% of all maternity staff in the hospitals. The aim of this follow-up study was to identify the key components that influence the sustainability of PROMPT in the Philippines, a middle-income setting, using focus group methodology.

## Methods

This was a 2 year project with seven maternity units participating in the Philippines PROMPT Project. Three units were selected for the qualitative study. This allowed for a representative sample of the seven units. The number of units interviewed was limited due to limited research resources. The units were purposively sampled (Table 1) as a representative sample of private, public and teaching hospital settings. The researchers planned to include additional units if data saturation was not achieved. However this was not required.

**Table 1:** Demographic details of the hospitals selected for this qualitative study

Unit	Public or Private	Department of Health governed	Location	Average number of births per year	Number of local PROMPT courses	% staff trained
A	Private	No	Manila	< 4000	4	80
B	Public	No	Manila	4000	6	93
C	Public	Yes	Manila	> 10,000	5	97

Focus group participants were recruited by distributing study information leaflets to all staff undergoing training in each unit, aiming for six to eight multi-professional volunteers for each focus group. Staff interested in participating were asked to contact the local PROMPT trainers. Local PROMPT trainers were responsible for ensuring multi-professional representation (Table 2). Focus groups were conducted rather than individual interviews, to reflect the multidisciplinary nature of the training.

**Table 2:** Number of participants in each focus group (FG) by staff group

Staff Group	Unit A: FG1	Unit A: FG2	Unit B: FG1	Unit B: FG2	Unit C: FG1	Unit C: FG2
Obstetric Nurse	5	3	0	6	1	2
Obstetric Resident	1	1	3	3	2	3
Obstetric Consultant	2	1	3	0	2	2
Anaesthesia Resident	0	0	0	0	0	4
Anaesthesia Consultant	1	1	0	0	1	0
Total	9	6	6	9	6	11

*FG1* Focus Group 1 comprising PROMPT trainers, *FG2* Focus Group 2 comprising participants of the local PROMPT training

All focus group participants gave written consent. Two focus groups were held in each unit in September 2016. The trainers and training participants were interviewed in separate focus groups to allow more open discussions amongst the groups.

- Focus Group 1 comprised local PROMPT trainers
- Focus Group 2 comprised local PROMPT training participants.

KG, the lead researcher of the Philippines PROMPT Project, facilitated the focus group discussions using a semi-structured topic guide [see ‘Supporting Information: Topic Guide Facilitators, Topic Guide Participants’]. Topics relevant to the group were used for discussion. This topic guide was adapted from a topic guide developed for and piloted in a parallel-process evaluation of PROMPT training in Scotland. ML, a Research Midwife, made field notes to record key phrases and non-verbal communications. Nobody else was present other than the researchers and participants. The focus group participants were aware that KG and ML were female PROMPT faculty members from the United Kingdom but did not know any personal information. KG and ML had prior experience of focus groups and Qualitative Research Methodology. Focus groups were conducted in English and lasted 25–54 min and were audio recorded. All participants were fluent in English. All the training resources were in English. Transcripts were produced by an independent transcription company. Transcripts were not returned to participants for comments or correction and the participants were in agreement with this plan. NViVo 10 software (QSR International) was used to manage the data. Reflexive thematic analysis process was used for analysis. Transcripts were reviewed and initial codes were developed by KG. Three researchers independently coded the same transcript and compared the codes. Any discrepancies were addressed by identifying the code that most suited the research question. This process was carried out for three transcripts until consistency of interpretation was achieved. The codes from the complete dataset were collated. Codes that occurred more frequently or were infrequent but felt to be significant to the study question were identified and initial themes were identified. These themes were refined through an iterative process to identify the best fit for themes that had an influence on sustainability of the project [28]. The qualitative researcher and RB had not been involved in delivering the training bringing a more independent interpretation of the data.

## Results

The focus groups comprised multidisciplinary participants including obstetric nurses (17), resident obstetricians (13), consultant obstetricians (10), resident anesthesiologists (4) and consultant anesthesiologists (3). All the participants had attended the PROMPT training that was delivered in English and had good spoken English. The facilitators were familiar with working together within their teams and therefore all contributed freely to the focus group discussions. Within the training participants’ focus groups, not all staff were familiar with working together and the consultant obstetricians and anaesthesiologists were notably more forthcoming during the discussions. A conscious effort was made to involve all participants, encouraging them to give their views and ensuring multidisciplinary representation.

**Fig. 1:**
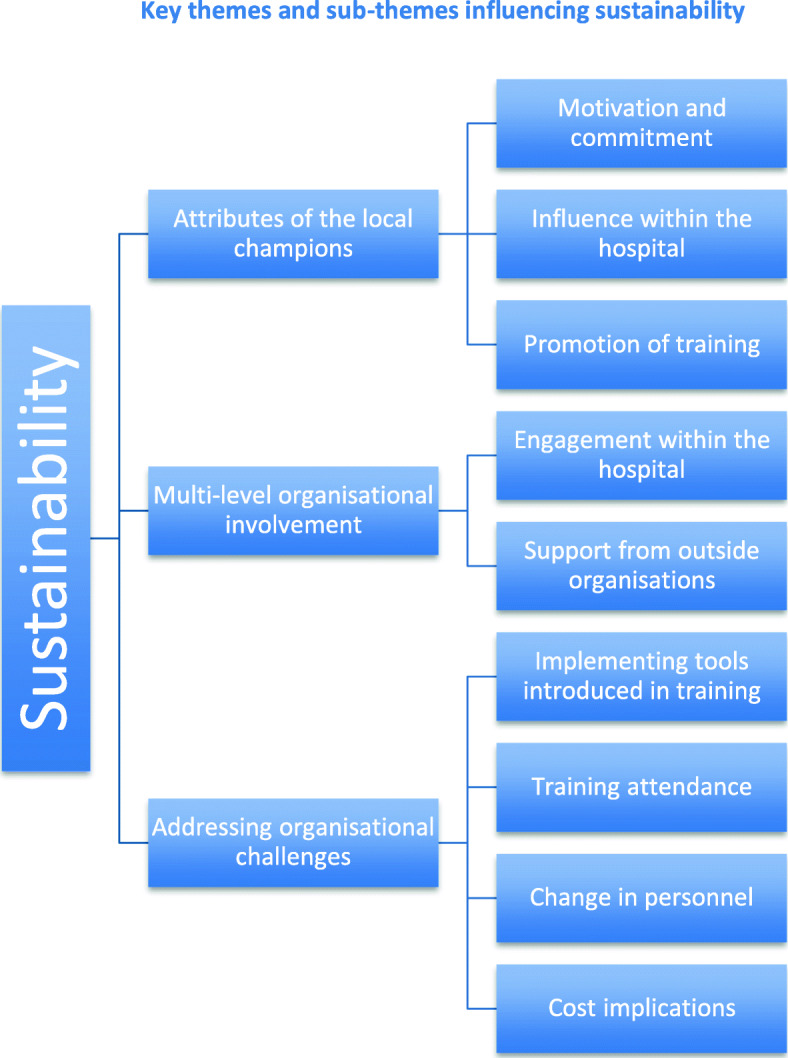
Key themes and sub-themes influencing sustainability

Three main sustainability themes and nine sub-themes were identified (Fig. 1). These were: attributes of the local champions, multi-level organisational involvement and addressing organisational challenges.

### Theme 1: attributes of the local champions

**Fig. 2:**
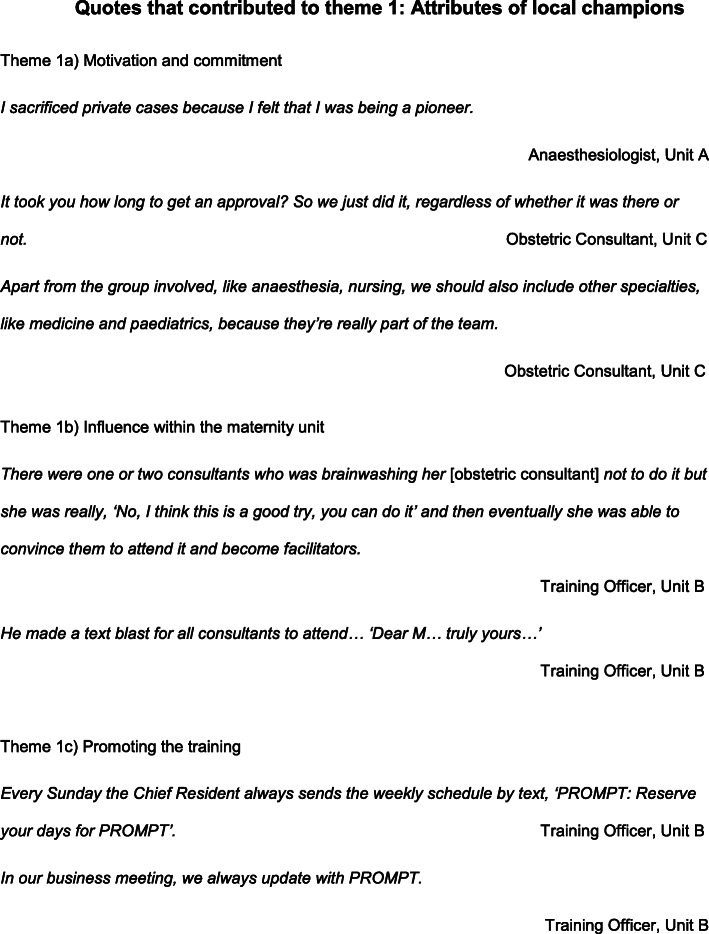
Quotations contributing to the formation of Theme 1: *Attributes of local champions*

Implementing the Philippines PROMPT Project required clinical leads from the participating hospitals to nominate a team of local champions responsible for coordinating and running the local PROMPT training in their maternity units. Each hospital had five multi-professional local PROMPT champions, representing each staff group contributing to maternity care: Obstetric consultant and resident, Obstetric nurse, Anesthesiologist and Training Officer. Three key sub-themes related to local champions emerged from the focus groups: motivation and commitment, their influence within the hospital and how they promoted the training. (Fig. 2).

#### Motivation and commitment

Driven by their belief in the value of the training, the champions demonstrated their commitment by meticulously organising the training days, rescheduling their clinical commitments and even sacrificing other incentives. Where there were challenges to seeking approval from hospital committees, the champions showed their commitment by persistence and using their initiative outside of the organisational process. The champions not only planned how to deliver the training, but also considered additional service improvements and expansion of the training to include other specialties.

#### Influence within the maternity unit

The champions were often respected local leaders who could influence their colleagues within the maternity department and engage senior staff in the training, particularly senior consultants, some of whom were initially reluctant to attend. In two units, local champions were able to engage senior managers to support the training. In one unit, the hospital chairman promoted the training by sending personalised text messages to their consultant colleagues.

#### Promoting the training

The champions took initiative and used various techniques to promote training participation. These included regular updates at staff meetings, texts and innovative publicity materials such as T-shirts designed with the Project logo.

### Theme 2: multi-level organisational involvement

**Fig. 3:**
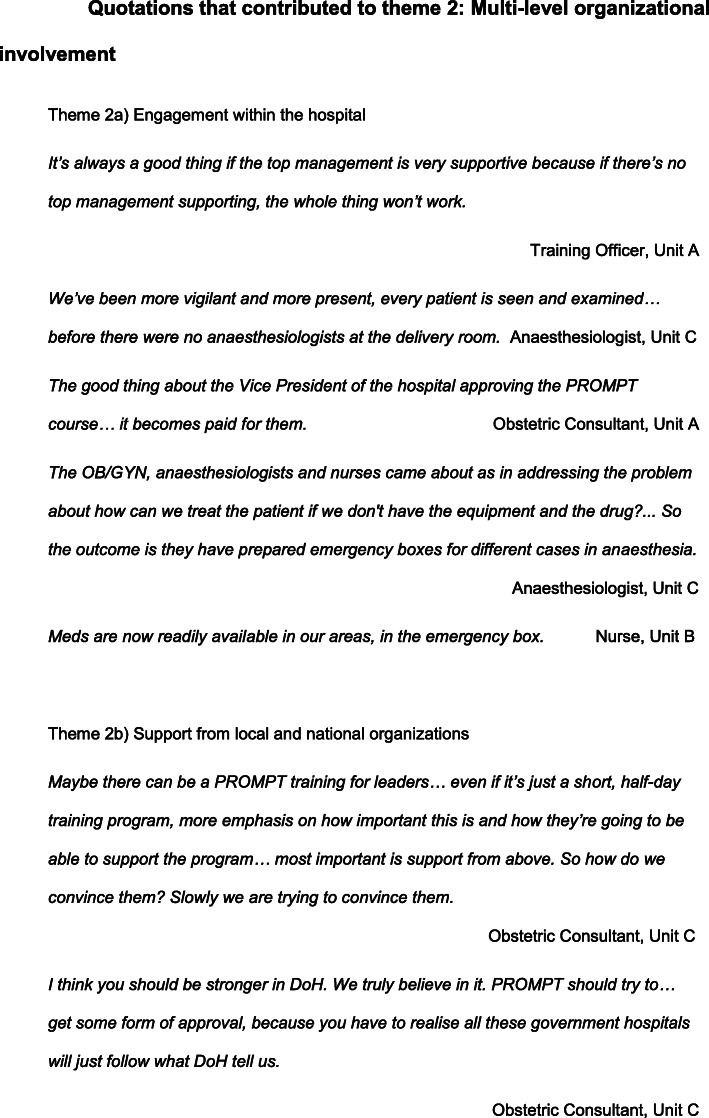
Quotations contributing to the formation of Theme 2: *Multi-level organisational involvement*

Another theme that emerged from the focus groups was multi-level organisational involvement, which included clinical leads of the specialty involved, as well as members of the Hospital Trust board, clinical support services, support from local policy makers and endorsement from national organisations. (Fig. 3).

#### Engagement within the hospital

Multi-level involvement within the hospital enhanced the implementation process and led to local service improvements that could be employed to further reinforce the perceived utility of the training. Examples of successful initiatives included: organisation of emergency equipment into emergency boxes and allocation of a dedicated anesthesiologist to the labour ward, ensuring readily available anaesthetic expertise. Support from the senior leadership team, such as Obstetrics Department Leads, seemed to validate the value of training to staff and provided useful role modelling. Senior leadership incentivised staff to attend by ensuring that the training occurred during paid working hours.

Multi-level support from clinical services not directly involved in maternity care was improved after local PROMPT training and helped to create sustainable service improvements. For example, staff attending training identified that some medications required in an emergency were not readily available as they had to be prescribed and dispensed off-site, and this caused delays in providing potentially life-saving treatment. The problem was conveyed to the pharmacy department and a system was organised so that these medications were stored on the Labour Ward, replacing the previous off-site dispensing system.

#### Support from local and national organisations

Each focus group identified the benefit of official support from local policy makers and national organisations such as Department of Health (DoH) or Philippine Obstetrical and Gynecological Society (POGS) mandating the training. They suggested that embedding the training into the DoH national curriculum would provide traction for all DoH-governed hospitals to implement the training. A mandate from POGS would call obstetricians to action although potentially may not appeal to non-obstetricians, contrary to the multi-professional nature of PROMPT training. Unit C, who had relied mainly on the resourcefulness of the champions to implement the training in the absence of the Medical Director’s support, suggested ideas for involving and getting traction with local leaders.

### Theme 3: addressing organisational challenges

**Fig. 4:**
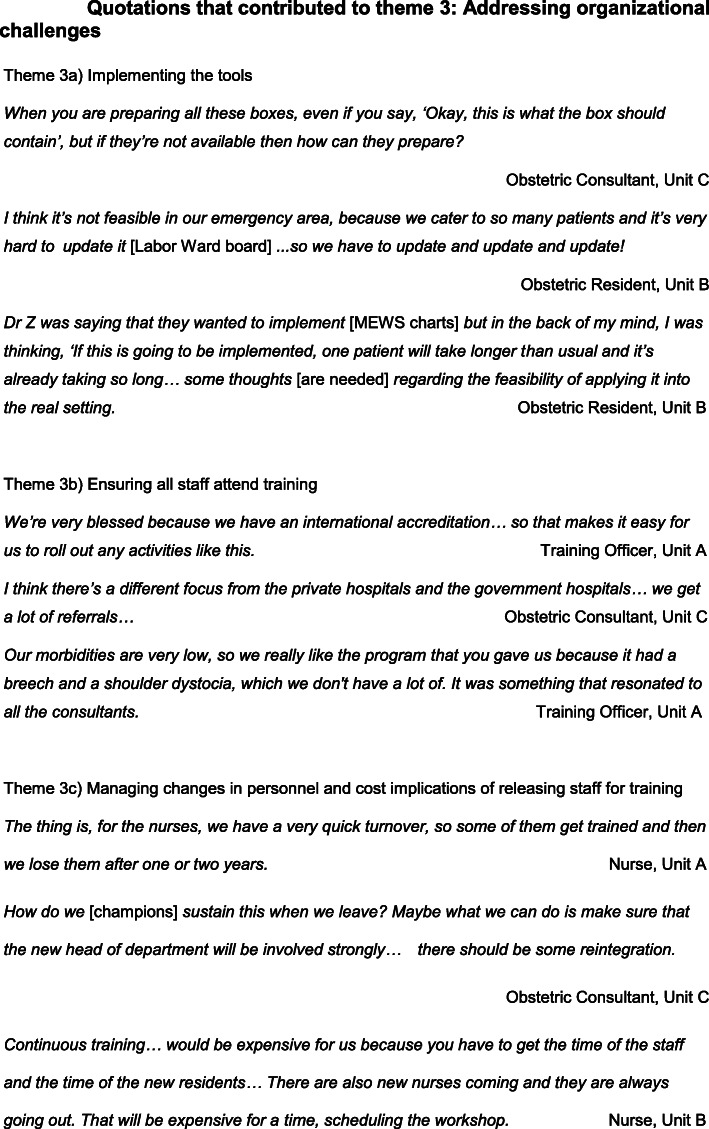
Quotations contributing to the formation of Theme 3: *Addressing organisational challenges*

Each hospital identified similar, and apparently universal, challenges to sustainability: promotion, dissemination and adoption of the tools introduced through the training, securing staff attendance on the training days, managing changes in staff personnel and the cost implications for releasing staff for training. (Fig. 4).

#### Implementing the tools

During the training, tools such as a Labour Ward Board, a Maternity Early Warning Score (MEWS) chart and emergency boxes for clinical emergencies such as pre-eclampsia and postpartum haemorrhage were introduced. These practice tools were extremely popular and although all of the trainers wanted to implement these tools, they recognised challenges with their availability and funding. MEWS charts utilise a red and amber warning score system to aid early recognition of the unwell woman [29] but staff were concerned that colour printing these single-use charts would incur significant costs. Emergency boxes organise key equipment, drugs and algorithms required for specific emergencies into accessible, portable boxes. Some clinicians questioned how the replenishment of supplies would be funded and who would be responsible for refilling the boxes after each use.

Although senior staff were keen to implement the tools, at ground level some Obstetric residents expressed concerns that high patient volume and heavy workload impacted upon the time available to implement these tools during routine clinical practice.

#### Ensuring all staff attend training

The focus groups reported challenges with local staff attending training due to staffing pressures restricting access to protected study leave, staff rotation in and out of the maternity departments, and securing the attendance of staff that had initially signed up to attend training.

In Unit B, only 1 anesthesiologist attended the training due to insufficient staffing to cover clinical commitments. Unit C staff were often requested to leave the training early to fulfil clinical demands. Unit C suggested restructuring the training day into two half-days to reduce the length of time that staff were away from the wards in a single session, therefore reducing the likelihood of staff being removed from training.

Differences in public and private hospital challenges were apparent. Unit A, as a private hospital, did not receive emergency patient referrals and had an international accreditation that enabled training to be embedded into the departmental policy. Units B and C, as tertiary public hospitals, received emergency patient referrals from outlying units, therefore patient volume, workload and staff availability for training could be unpredictable. Unlike Unit C, Unit B is a University teaching hospital with a structured programme for Training and Research that facilitated the implementation process.

Unit A staff explained that they infrequently experience obstetric emergencies; this is likely related to fewer births and patient selection. Unit A recognised the need for their staff to attend training despite experiencing fewer morbidities and mortalities compared to public hospitals. They valued the training as an opportunity for their staff to refresh these less frequently used skills, which may improve clinical outcomes.

#### Managing changes in personnel and the cost implications of releasing staff to attend training

Suggestions to manage changes in personnel and regular staff rotations included recruiting more trainers to build a larger faculty and running more local PROMPT courses, although the cost implications of releasing staff to attend further training was highlighted.

Suggestions to improve attendance included making training mandatory for all staff and providing regular outcome-based feedback to demonstrate clinical improvement to encourage ‘buy-in’ from the staff, particularly those who may not perceive the training as valuable.

## Discussion

Three key themes influencing sustainability were identified by the focus group participants: attributes of local champions, multi-level organisational involvement and addressing organisational challenges.

These themes were universal across all three hospitals and agreed amongst the individual staff groups. The themes resonate with findings from studies in high and low-income settings [10, 11, 30]. Similar challenges and facilitators were identified, such as staffing and funding resources and involvement of senior leadership and local policy makers. Our study adds the perspectives of local maternity unit staff to the current literature on sustainability of healthcare interventions, provides an exemplar of implementation and associated challenges within middle-income settings and outlines potential strategies to improve sustainability of local training.

Adoption of an intervention in one setting may not be generalisable to other settings [1]. There is a call for a deeper understanding of the underlying social processes and ‘active ingredients’ supporting implementation [31]. This unit-level study directly explores ground-level staff experiences of the implementation process. These staff are immersed in their own context and most likely to understand issues specific to their unit. They also have an integral knowledge of the staff and system that external organisations may struggle to acquire. This study highlights the advantages of harnessing local expert knowledge to select influential champions to lead implementation of the intervention and address local challenges with effective strategies. The local champions were the driving force of the training, and apparently key to the success of initial implementation, as well as sustainability. There was no specific methodology to objectively assess the suitability of the champions, however this subjective system of the unit leaders selecting the champions appears to have been successful. Further research is indicated to identify this intuitive selection of champions as this is likely to have an impact on the implementation of the project.

Implementation is influenced by multiple interacting factors at numerous levels within, and across, healthcare systems [12]. In this study, backing from senior leaders within the hospital setting was crucial for embedding the training into hospital policy. Their perceived involvement was not essential for implementation but it was evident in Unit C that without this support, greater efforts were required from the champions to implement the training. Involvement from other clinical services, such as the pharmacy department, enabled service improvements that enhanced the perceived value of the training and reinforced the importance of it being locally run. A national mandate for training was recommended, particularly by Unit C. However, some studies suggest that top-down approaches can fail to engage ground-level staff who may not perceive the value of the intervention or may feel change is being imposed upon them [32]. Tailoring implementation to an organisation’s needs increases the likelihood that staff will adopt the intervention [33]. This study demonstrates a bottom-up approach utilising the expertise of local staff to mobilise ground-level staff and promote local ownership, with support from external organisations to enable scale-up and sustainability.

All hospital staff groups described similar challenges regarding staffing and resources and presented different strategies for overcoming them. Increasing the number of training faculty can build a critical mass of trainers sufficient to maintain sustainability beyond any changes in training personnel. Regular knowledge and outcome-based feedback was considered as a method to validate the training and encourage ‘buy-in’ from any reluctant staff, as well as policy makers. Ground-level staff anticipated difficulties implementing practice tools due to high patient turnover, time pressures and restricted funding. This study demonstrates that the feasibility of introducing these tools should be tested, possibly with a short-term service evaluation that includes feedback from staff on their perception of these tools. This process could guide the successful integration of tools that support local clinical practice.

Many of our findings align with the current literature, [34–37] which is positive and may validate our methodology and findings, however we have also identified some issues specific to middle-income settings. The three hospitals are representative of all tertiary maternity care settings in the Philippines: private, public and university-affiliated tertiary urban hospitals, and we would expect our findings to be representative of other similar-sized hospitals with comparable demographics in other middle-income settings.

The limitations of the study were acknowledged and addressed where possible. The team was aware of possibilities of reporting bias and this was reduced by conducting separate focus groups for trainers and participants to reduce any reporting bias. The focus group facilitators were involved in delivering the original PROMPT Train-the-Trainers (T3) programme to the participating units, and as a consequence, their involvement may have introduced a bias. Although Focus Group 1 participants were aware that the focus group facilitators were part of the original T3 training team, Focus Group 2 participants had not attended the original T3 programme and were not aware of the focus group facilitators’ involvement. The scope of the focus groups was explained to the participants and they were encouraged to give honest opinions. The participants had good spoken English but we cannot exclude the possibility of losing subtle findings in translation. Field notes were used to capture the non-verbal interactions. It is debatable whether the effect of using the same team to run focus groups was entirely detrimental to the quality of data. The team were immersed in the setting up of the training programme and this familiarity enabled them to explore issues that an independent researcher might not have.

These results may not be representative of smaller non-tertiary or rural hospitals, where the infrastructure and systems are different to the study hospitals from large metropolitan settings. However, the findings from this study are likely to be relevant to future sustainability of local training across a variety of hospital settings. A follow up of these focus groups after a period of time would be useful to identify longer term sustainability of the project and highlight new themes.

## Conclusions

To conclude, we have identified three key themes that influenced the sustainability of a local obstetric emergencies training programme in a middle-income setting. These factors, including potential barriers to sustainability, could be usefully considered when designing and implementing training programmes in other middle-income settings. Local champions are vital to identifying challenges and develop strategies specific to their organisation. Successful strategies could be collated and made available to local training teams and across networks. In addition, policy makers and major stakeholders should be engaged early on, to gain support for funding and to align training with local or national training priorities.

## Supplementary Information

**Additional file 1.** Topic Guide Facilitators.

**Additional file 2.** Topic Guide Participants.

## Data Availability

The datasets used and/or analysed during the current study are available from the corresponding author on reasonable request.

## Abbreviations

DoH: Department of health
MEWS: Maternity early warning score
POGS: Philippine obstetrical & gynecological society
PROMPT: Practical obstetric multi-professional training
