# Supplementary material for: Key components influencing the sustainability of a multi-professional obstetric emergencies training programme in a middle-income setting: a qualitative study
Source: BMC Health Serv Res. 2021 Apr 26;21:384. doi: 10.1186/s12913-021-06385-5 (PMC8077832; doi:10.1186/s12913-021-06385-5)
Supplement: Supplementary file 1 — Additional file 1. Topic Guide Facilitators. [file 12913_2021_6385_MOESM1_ESM.docx]

**FOCUS GROUP TOPIC GUIDE: LOCAL PHILIPPINES PROMPT FACILITATORS**

**Introduction**

- Thank you for meeting with us today, we really appreciate you sparing your time. Thank you for your continued enthusiasm for the Philippines PROMPT Project.
- The aim of today’s focus groups is to explore your experiences of setting up PROMPT in your unit and to understand how the training has been implemented. This will help us to improve our training and guide further implementation.
- This is not an assessment of the unit or of you or your staff. There are no ‘right’ or ‘wrong’ answers.
- We do audio record and transcribe the discussions. Mary is here as a scribe.
- All information is treated in confidence and will remain anonymous. The discussions will remain confidential within this group and will not be discussed outside of this group.
- Have you read and understood the information sheet? Do you have any questions?
- Participation is voluntary. If you are happy to participate, please complete the consent forms.

**INTRODUCTORY QUESTIONS**

- Names and roles
- How long have you worked here?

**IMPRESSIONS OF T3 TRAINING**

- Had you heard of PROMPT prior to attending the Train-the-Trainers programme in Manila? What did you know about it?
- What were your first impressions of the PROMPT Train-the-Trainers programme?
- Was the PROMPT training similar or different to other training you might have received/been involved with? If so, how?

**IMPLEMENTATION: HOW IS TRAINING DELIVERED?**

- How did you set up training your team?
- How many planning meetings did you have before the first training day?
- Are any of you already trainers for other training programmes?
- Did you use any of the resources in the DVD and course manual? What was most useful? Was there anything you didn’t use?
- Have you introduced any of the tools during the training days: Labour Ward board, MEWS charts, emergency boxes, algorithms? Are any of these being used in clinical practice?

**IMPLEMENTATION: WHAT IS DELIVERED?**

- You have run *x* number of training days so far. How did you find trying to set up these training days? Any particular barriers or facilitators?
- How do you structure the training days?
- Have you changed or adapted anything from the training day or course manual to suit your own unit?
- Did you feel there were some things that you felt were important to keep the same?
- Did you have support from hospital management and the Medical/Clinical Director?
- How do you feel the maternity staff responded to the prospect of training days?
- On average, how many people attended each training day? How have you managed to release staff to attend the training?
- Was there a mix of Obstetricians, Obstetric Nurses, Anesthesiologists and Nursing Attendants?
- Were any groups of staff easier or harder to recruit (eg. residents/consultants, anes) Why?

**IMPACT**

- Do you feel that the participants have been engaged with PROMPT? What makes you think that?
- Did any changes come about as a result of introducing PROMPT? eg. changes in clinical practice or system processes
- What feedback have you had so far? eg from evaluation sheets
- What effects, if any, do you think PROMPT has had here?

**CONTEXT**

- What’s it like working here? What do you think about the safety culture of the unit?
- Do staff tend to stay here for many years or move on?

**GENERAL FEEDBACK**

- How easy has it been to set up training? What has helped with implementing it? eg. the local & PMF team support
- Have you found anything difficult? Can you say why?
- How have you tried to encourage or facilitate implementation of local training here?
- Can you think of anything that would have helped you implement training more easily?
- What do you think overall about PROMPT? Does it make sense?
- Do you think PROMPT has become a normal part of practice for the unit?
- What are your future plans for PROMPT training?
- Do you foresee any problems sustaining training?
- Can you think of anything that may help with sustaining training?
- Do you have any additional comments?

Any other questions or feedback?

Thank you for your time and for sharing your views. This focus group discussion will remain confidential and anonymous.
