# Supplementary material for: Key components influencing the sustainability of a multi-professional obstetric emergencies training programme in a middle-income setting: a qualitative study
Source: BMC Health Serv Res. 2021 Apr 26;21:384. doi: 10.1186/s12913-021-06385-5 (PMC8077832; doi:10.1186/s12913-021-06385-5)
Supplement: Supplementary file 2 — Additional file 2. Topic Guide Participants. [file 12913_2021_6385_MOESM2_ESM.docx]

**FOCUS GROUP TOPIC GUIDE: LOCAL PHILIPPINES PROMPT TRAINING PARTICIPANTS**

**INTRODUCTION**

- Introduce KG and ML. Thank you for coming today and sparing your time. We really appreciate it.
- Explain Philippines PROMPT Project. This unit has been implementing local PROMPT training since *xxxx* 2015/16. The aim of this focus group is to explore your experience of attending a local PROMPT training day and to understand how PROMPT has been implemented in this unit. We are also conducting a focus group of the facilitators. We hope that the information from these focus groups will help us to improve our training and inform further implementation, hopefully improving maternity care for mothers and their babies.
- This is not an assessment of the unit or the staff. There are no ‘right’ or ‘wrong’ answers.
- We audio-record the discussions and transcribe anonymously. ML is scribe.
- All information is treated in confidence. The discussions will remain confidential within the group and will not be discussed outside of the group.
- Have you read the information sheet?
- Do you have any questions?
- Participation is voluntary. If you would like to participate, please complete the consent forms.

**INTRODUCTORY QUESTIONS**

- Introductions: names and roles
- How long have you worked here?

**IMPRESSIONS OF LOCAL TRAINING**

- Had you heard of PROMPT before you attended the training?
- Have you attended any other maternity emergency training? Was that locally in your unit or somewhere else? Was this similar or different?
- How easy was it for you to attend training?
- Did you attend training on a day off or a working day?
- What were your first impressions of the PROMPT training?
- How did you find training in multi-professional teams? Was that a new way of training for you? What did you think about that?
- How did you feel doing simulation training?
- Were there things you liked about PROMPT? Why?
- Do you feel anything could be improved? Why?
- Were there any discussions during the training eg. debrief or feedback? Did you feel able to contribute to these discussions?
- Did you receive any course materials? What did you think about them?

**CONTEXT**

- What is it like to work here? eg. workplace culture
- Has anything noticeably changed here since training was introduced?
- Have you noticed any new tools? eg. Labour Ward board, guidelines, boxes, algorithms, MEWS charts

**GENERAL FEEDBACK**

- What do you think overall about PROMPT?
- Does it make sense?
- Has PROMPT become a normal way of practice for you?
- Can you think of anything that would make the training better?
- Do you have any additional comments?

**BREAKOUT SESSION**

Doctors and nurses to be divided into two groups to explore the multi-professional working theme.

- How did you feel working with doctors/nurses?
- Had you trained with doctors/nurses before?
- Would you feel more comfortable to prompt doctors/nurses in clinical practice or emergencies since this training?
- Do you think you will feel more comfortable working with doctors/nurses in general?
- Any other comments about multi-professional working?

Thank you for your time and for sharing your views. All information will remain confidential and anonymous.
